# Monitoring BMI-dependent gait adaptations through smart insole technology: implications for musculoskeletal health

**DOI:** 10.1186/s13102-026-01703-y

**Published:** 2026-05-01

**Authors:** Xue Liu, Xinyu Dou, Yong Wang, Yan Yuan, Fansen Wei, Chaobing Zhang, Chenyi Zuo, Jingsong Mu

**Affiliations:** 1 Graduate School, Bengbu Medical University, Anhui Bengbu, 233000 China; 2 College of Mechanical Engineering, Hefei University of Technology, Hefei, 230009 China; 3 Department of Rehabilitation Medicine, The Third People’s Hospital of Hefei, Anhui Hefei, 230022 China; 4 School of Smart Wellness, Anhui Vocational College of City Management, Anhui Hefei, 230011 China; 5 Department of Rehabilitation Medicine, The First Affiliated Hospital of USTC, Division of Life Sciences and Medicine, University of Science and Technology of China, Anhui Hefei, 230000 China; 6 Department of Rehabilitation Medicine, The First Affiliated Hospital of the China University of Science and Technology (Anhui Provincial Hospital), Anhui Hefei, 230000 China

**Keywords:** Body Mass Index (BMI), Gait Analysis, Smart Insoles, Plantar Pressure, Musculoskeletal Health, Young Adults

## Abstract

**Background:**

This study aims to identify differences in specific gait parameters (spatiotemporal kinematics, plantar pressure, and center of plantar pressure indicators) among young adults with different body mass index (BMI) levels, clarify their linear and nonlinear correlation patterns with BMI, provide a theoretical basis for the prevention and intervention of musculoskeletal injuries associated with abnormal BMI, and verify the practical value of a self-developed smart insole in gait monitoring.

**Methods:**

A total of 62 college students aged 18–28 years were enrolled and categorized into four groups (underweight, normal-weight, overweight, obese) based on the 2024 Guidelines for the Prevention and Control of Overweight and Obesity in Chinese Adults. All participants wore smart insoles and completed a 10-meter walking test. Twenty-one key gait parameters were extracted, including general gait parameters, gait cycle-related indicators, ankle angle indicators, five-division peak plantar pressure (PPP), and center of plantar pressure (COP)-related indicators. Inter-group differences were compared using one-way ANOVA or Kruskal-Wallis H test with Bonferroni correction, and correlations between gait parameters and BMI were analyzed via Spearman correlation analysis. Nonlinear relationships were explored using parabolic regression for parameters without significant linear correlation. Effect sizes (f = 0.40 for ANOVA, ε² = 0.398 for Kruskal-Wallis H test) were calculated based on primary outcome variables.

**Results:**

Analysis of 21 gait parameters revealed that 12 indicators were linearly correlated with BMI, while 6 showed a nonlinear (curvilinear) relationship with BMI. Significant inter-group differences (*p* < 0.05) were observed in relative stride speed (RSS), percentage of single support time (%SST), percentage of swing-phase time (%SPT), and peak pressures at the medial metatarsal (PPP-M), arch (PPP-A), and heel (PPP-H) regions, with large effect sizes indicating substantial practical differences. Specifically, RSS, %SST, and %SPT progressively decreased, while PPP-M, PPP-A, and PPP-H progressively increased across the four BMI groups (*p* < 0.001 after Bonferroni correction). The smart insole system accurately captured these differential parameters with high sensitivity and dynamic response performance.

**Conclusion:**

This study reveals the specific gait adaptation mechanisms of young people at different body mass index (BMI) levels. The self-developed smart insole can effectively capture these key differential parameters, providing a feasible quantitative technical method for the preliminary screening of gait abnormalities related to BMI and the monitoring of the musculoskeletal and motor health status of young people similar to the study cohort.

**Trial registration:**

This study was registered at the Chinese Clinical Trial Registry (Registration number: MR-34-23-032327) on September 8, 2023.

**Supplementary Information:**

The online version contains supplementary material available at 10.1186/s13102-026-01703-y.

## Highlights

Innovative Technology Application: The smart insole technology integrated with pressure and motion sensors is applied to collect gait parameters of young adults with different BMI levels, breaking through the limitations of traditional laboratory-based gait analysis methods.

Key Gait parameters Identified: The preliminary analysis results showed that there were significant statistical differences in relative step speed (RSS), percentage of single support time (%SST), percentage of swing phase time (%SPT), PPP of the medial metatarsal (PPP-M), PPP of the arch (PPP-A), and PPP of the heel (PPP-H) among the four groups. Such differences were difficult to detect with previous devices but can be accurately captured by smart insoles.

Filling Research Gaps: It addresses the issue that subtle adaptive gait changes in young overweight/underweight populations have been overlooked in existing studies, providing potential early markers for musculoskeletal dysfunctions associated with abnormal BMI. It also verifies the application potential of smart insoles in the early screening of BMI-related gait abnormalities.

**Supplementary Information:**

The online version contains supplementary material available at 10.1186/s13102-026-01703-y.

## Introduction

The continuous rise in obesity has become a major challenge threatening public health. *The 2025 World Obesity Map* [1] shows that it is estimated that by 2030, the number of adults with high BMI worldwide will exceed 2.9 billion. BMI is an effective indicator reflecting the degree of obesity and the physical health [2]. Abnormal BMI not only increases the risk of developing a variety of diseases [3], but also causes persistent damage to the motor system by altering the force-bearing pattern [4]. On the other hand, individuals with low BMI often face the problem of insufficient muscle strength, which increases the risk of injuries during exercise [5]. However, relying on their strong neuromuscular plasticity, young adults can develop unique compensatory mechanisms to maintain motor function when coping with different loads [6].

As the most basic motor function of the human body, walking can sensitively reflect the load status of the lower limbs and the health of the joints [7]. At present, laboratory gait analysis technologies, such as pressure plates [8] and infrared camera systems [9], can provide high-precision gait data in specific environments, but they generally face bottlenecks such as high environmental dependence and limited dynamic tracking capabilities. With technological iteration, wearable devices (such as plantar pressure sensors [10], surface electromyography sensors [11], and inertial sensors [12]) have broken through spatial limitations and realized daily dynamic monitoring, but they face new challenges such as single-dimensional data acquisition and insufficient multi-modal fusion.

As a new type of wearable device, smart insoles provide an innovative technical approach to addressing the above-mentioned issues. Integrated with high-precision pressure sensors and inertial measurement units, this device breaks through the limitations of laboratory environments, enabling continuous collection of gait data in real-life scenarios such as daily walking, climbing up and down stairs, and outdoor activities, thus effectively avoiding data bias caused by the Hawthorne Effect. It can simultaneously acquire kinematic and dynamic indicators, construct individualized “gait fingerprints”, and provide multi-dimensional data support for comprehensive analysis of gait characteristics [13].

This study adopted the smart insole system independently developed by our team [14], with two core objectives: First, to systematically explore the differences in key gait parameters (spatiotemporal parameters such as relative step speed and single support time ratio, plantar pressure indicators including peak pressure of medial metatarsal/arch/heel, and center of plantar pressure-related parameters) among college students with underweight, normal weight, overweight, and obese BMI levels, and clarify the linear and nonlinear correlation patterns between these parameters and BMI; second, to evaluate the performance of the self-developed smart insole in capturing subtle gait differences related to BMI, verifying its practical value in early screening of gait abnormalities and sports health monitoring. Through this research, we aim to fill the research gap in subtle gait adaptations of young people with abnormal BMI and provide a targeted theoretical basis for the prevention and intervention of BMI-related musculoskeletal injuries.

## Materials and methods

### Smart insole system

#### Technical architecture and performance analysis of smart insole

**Fig. 1:**
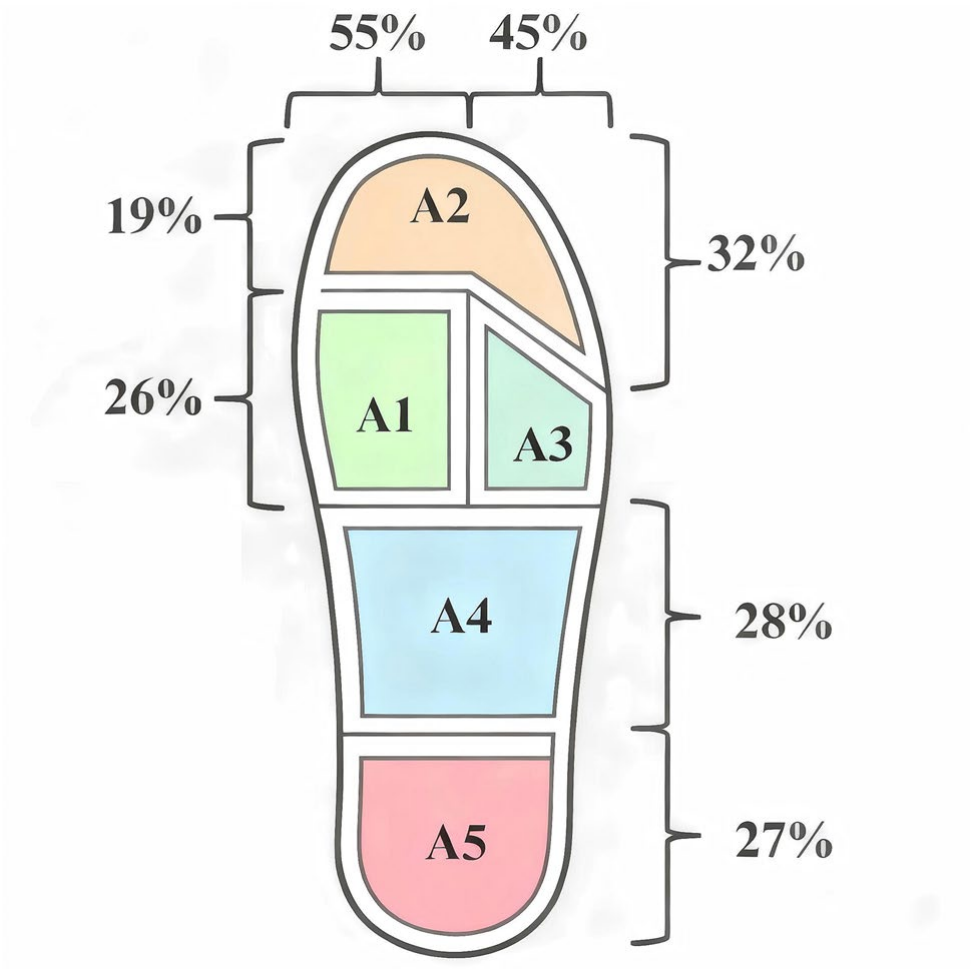
shows the percentage distribution of the five pressure-bearing areas on the insole along the longitudinal axis: the inner metatarsal zone (A1), the phalangeal zone (A2), the outer metatarsal zone (A3), the arch zone (A4), and the heel zone (A5)

The wearable smart insole developed in this study uses SHORE A30 silicone rubber as the base, integrates multiple sensors and a wireless transmission module, and forms a lightweight gait monitoring system. At the hardware level, the insole is divided into five pressure monitoring areas (Fig. 1). The plantar surface was segmented into five distinct regions (A1–A5) based on established anatomical landmarks and functional biomechanical principles. This segmentation strictly follows the osseous structure of the foot and the physiological weight-bearing distribution during gait:

- A1 (Medial Metatarsal zone): Defined anatomically by the heads of the 1st, 2nd, and 3rd metatarsals. Functionally, it is a critical weight-bearing region, particularly during the mid-stance phase, and is highly sensitive to alterations in body mass and balance control.
- A2 (Phalangeal zone ): Anatomically, this region corresponds to the distal phalanx. Functionally, it serves as the primary propulsion site during the terminal swing phase, generating the final push - off force for forward movement.
- A3 (Lateral Metatarsal zone): Encompasses the anatomical heads of the 4th and 5th metatarsals. Functionally, it acts as a lateral stabilizer, counteracting medial deviation of the center of pressure and providing structural support during weight transfer.
- A4 (Arch zone): Aligns anatomically with the medial longitudinal arch, spanning from the navicular bone to the base of the metatarsals. Functionally, it serves as the body’s primary shock absorber and spring mechanism, attenuating impact forces during heel strike and storing elastic energy for propulsion.
- A5 (Heel zone): Corresponding anatomically to the calcaneus (heel bone), specifically the posterior and inferior tuberosities. Functionally, it is the initial contact point during the gait cycle, absorbing the entire body’s impact force at heel strike.

Specifically, the segmentation boundaries between regions A1 to A5 were determined proportionally along the total longitudinal foot length (from the distal tip of the hallux to the posterior aspect of the calcaneus). The numerical values indicated (19%, 26%, 32%, 28%, 27%) represent the percentage of the total foot length occupied by each respective anatomical segment. This length-proportional division ensures consistent sensor positioning across different foot sizes while maintaining alignment with key weight-bearing anatomical landmarks. Each area is equipped with an RSCM17100KP201 barometric pressure sensor (a total of 5, with a full-scale accuracy of ± 1% to ± 2% and a resolution of 1 millivolt per unit) and an MPU6050 inertial measurement unit.

**Fig. 2:**
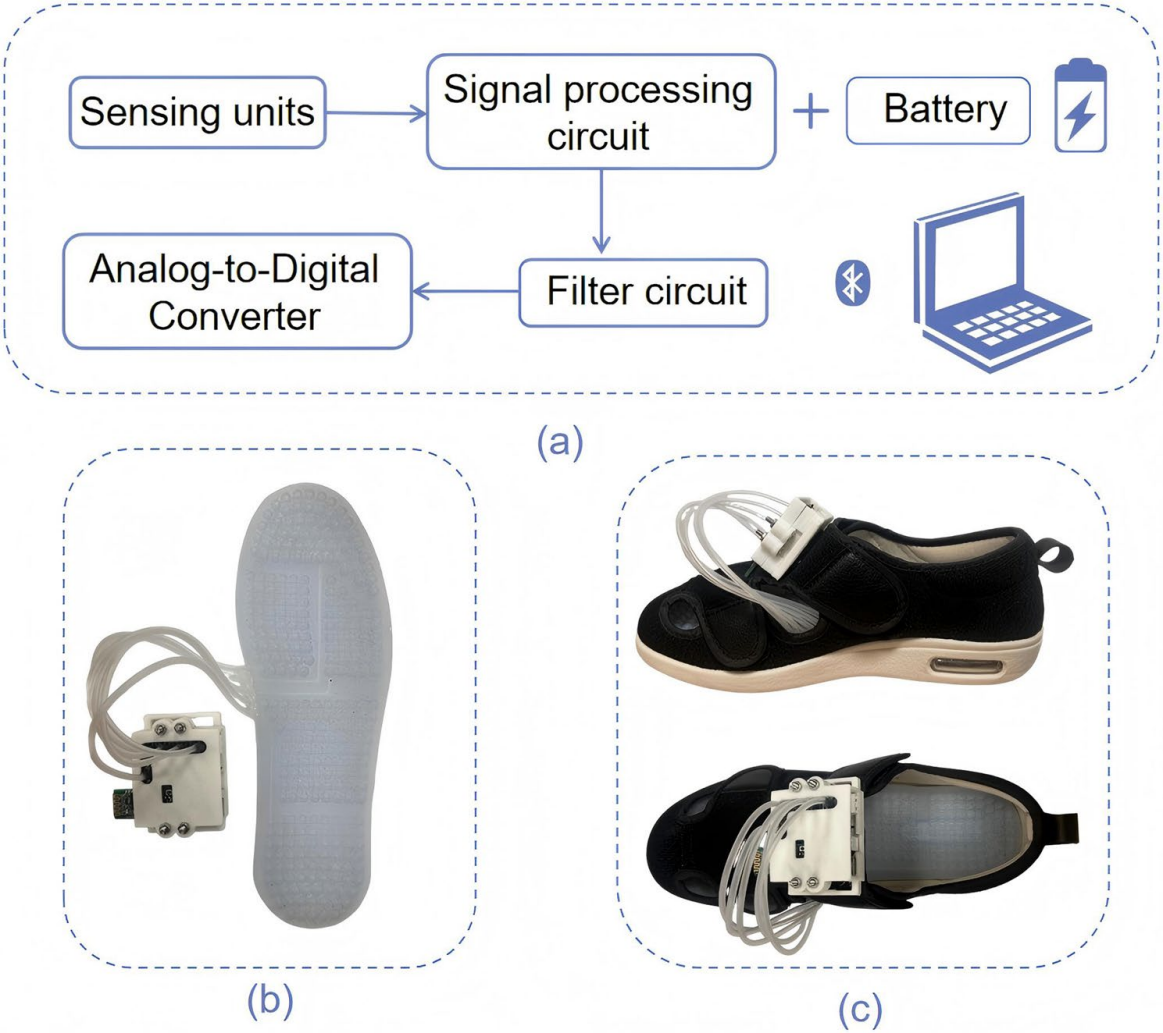
shows Hardware design and prototype of a smart insole system. **a** the hardware design principle, (**b**) the insole prototype, consisting of a sensing insole and a connected circuit module. **c** the assembly of the insole inside a shoe

The STM32 microcontroller is used to collect signals, which are then wirelessly transmitted to a PC via a Bluetooth module (HC05) (Fig. 2). The system is equipped with a 7.4 V/500 mA lithium battery, with a measured working current of 58 mA, which can operate continuously for about 8 h, meeting the needs of daily monitoring. Compared with the MS4525-1PSI insole system, the smart insole has *advantages in terms of sensitivity*,* nonlinear erro*r, hysteresis error, and response time [14, 15].

In terms of core performance, the self-developed smart insole was quantitatively compared with the commercial MS4525-1PSI insole system (a widely used device for plantar pressure and gait monitoring), and the results demonstrated significant advantages in key performance indicators related to gait parameter detection, with detailed performance comparisons and optimization amplitudes shown in Table 1.

**Table 1:** Performances comparison

Performance Indicator	MS4525-1PSI System	This Study’s Smart Insole
Sensitivity	-	3.63 mV/N
Nonlinear Error	4%	2.97%
Hysteresis Error	10%	3.37%
Accuracy Error	-	4.60%
Repeatability Error	5%	5.49%
Response Time	-	3.05 ms

#### Technical path and experimental verification of gait recognition

This insole integrates Ensemble Empirical Mode Decomposition (EEMD) with a Convolutional Neural Network-Long Short-Term Memory (CNN-LSTM) neural network to achieve high-precision gait analysis. During data preprocessing, a 4th-order Butterworth low-pass filter with a cutoff frequency of 30 Hz is used to clean the signals. By combining Kalman filtering to fuse IMU data, researchers calculate the foot attitude angles, extract 12 key features, and standardize them using Z-score. After decomposing the plantar pressure and attitude signals using EEMD, the 5th-order Intrinsic Mode Function with concentrated energy is selected as input to the model. The CNN-LSTM model extracts spatial features through two layers of 1D convolution (with 64/128 channels) and processes time series using an LSTM layer containing 128 hidden units. Previously, based on data from 20 subjects in 6 real-life scenarios (standing, turning around, walking, running, climbing up and down stairs), the team achieved a gait pattern recognition accuracy of 97.86%, a phase recognition accuracy of 97.24%, a Matthews Correlation Coefficient of 0.96, and a Root Mean Square Error of 0.15, which were significantly better than those of comparison models such as CNN and KNN [15].

### Participants

A total of 62 college students were recruited in this study (including 30 males and 32 females). The subjects participated in the experiment voluntarily. Referring to the standards in *Guidelines for the Prevention and Control of Overweight and Obesity in Chinese Adults (2024)* [16] the subjects were divided into 4 groups according to their Body Mass Index: Underweight group (BMI < 18.5 kg/m², *n* = 14), Healthy weight group (18.5 kg/m² ≤ BMI < 24.0 kg/m², *n* = 24), Overweight group (24.0 kg/m² ≤ BMI < 28.0 kg/m², *n* = 14), and Obesity group (BMI ≥ 28.0 kg/m², *n* = 10). It has obtained ethical approval from the First Affiliated Hospital of China University of Science and Technology (ethical approval number: 2023KY086). We ensured that all participants signed an informed consent form before the start of the study, clearly informing them of the way their data would be used. In addition, we anonymized the collected data and took stringent measures to ensure data security and privacy, complying with relevant ethical and legal regulations.

Inclusion criteria: (1) Age between 18 and 28 years old; (2) Voluntary participation in this study.

Exclusion criteria: (1) Physical Abnormalities: Students with a history of major diseases (such as cardiovascular diseases, neurological diseases, musculoskeletal diseases, etc.) and diseases or injuries that affect gait (such as arthritis, flat feet, pescavus, or a recent history of fractures or surgeries, etc.); (2) Mental and Psychological Disorders: Students with mental or psychological disorders who have difficulty cooperating to complete the test tasks; (3) Intention to Withdraw Mid-study: Students who clearly state that they are unable to continue participating in the study during the research process should be excluded.

Sample size determination was conducted retrospectively to validate the statistical power of the enrolled cohort, as a prospective sample size calculation was not performed prior to study initiation. The validation was based on the primary outcome variable (relative step speed ), which exhibited the largest effect size in preliminary analyses. For the one-way analysis of variance (ANOVA) and Kruskal-Wallis H test (for non-normal data), key parameters were set as follows: two-sided significance level (α) = 0.05, number of groups (k) = 4 (underweight, normal-weight, overweight, obese), and effect size (f = 0.40 for ANOVA, ε² = 0.398 for Kruskal-Wallis H test) derived from the actual study data. A total of 62 participants were enrolled, and post-hoc power analysis confirmed that the study achieved a statistical power (1-β) of 0.892 for the primary outcome, exceeding the conventional threshold of 0.80. This indicates that the sample size was sufficient to detect the moderate-to-large intergroup differences in key gait parameters observed in this study.

### Procedures

The test was conducted in the corridor of the Rehabilitation Department of the First Affiliated Hospital of the China University of Science and Technology. Before the formal test, the participants were strictly screened: they were required not to have consumed alcoholic beverages, sleeping pills, sedatives and other drugs within 48 h, and to avoid strenuous activities one day before the test, so as to ensure that their heart rate and blood pressure were in a stable state during the test.

**Fig. 3:**
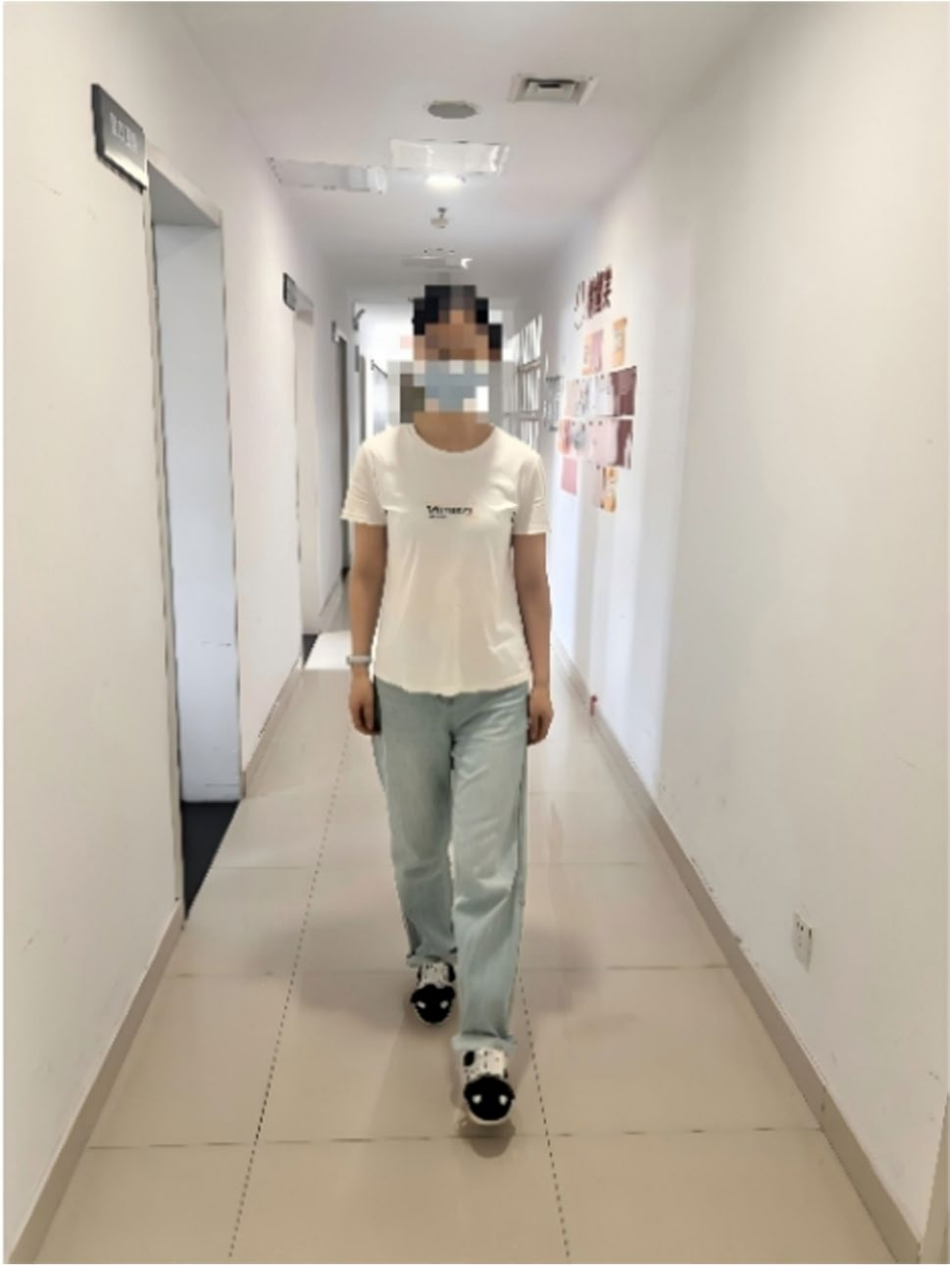
a picture of the subject testing the shoes with smart insoles in the hospital corridor

During the test, all participants were required to wear specially made insoles and perform a 10-meter walking test. When standing, they were asked to place their hands on both sides of their bodies, keep their feet shoulder-width apart, and maintain an upright and relaxed posture. Once this preparation phase was completed, participants were requested to walk along a 10 m long walkway at their self-selected speed in the most natural manner (Fig. 3). A total of 3 formal tests were conducted, with a 2-minute rest interval between each test. The testers recorded data. Finally, the average value of the 3 tests was used for statistical analysis.

### Research Indicators

The observation indicators in this study include.

- General gait parameters: Step length (SL), Step length ratio (SLA), Step speed (SS), Relative step speed (RSS), Cadence (Cad). SLA = SL/height [17], RSS = SS/height [18].
- Gait cycle-related indicators: Gait cycle time (GC), Percentage of single support time (%SST), Percentage of double support phase time (%DSPT), Percentage of swing phase time (%SPT).
- Ankle angle indicators: Foot angle: Step angle (SA), Maximum plantar flexion angle (MPFA), Maximum dorsiflexion angle (MDFA), Foot roll angle (FRA).
- Five-division Peak plantar pressure (PPP): PPP of medial metatarsal (PPP-M), PPP of phalangeal (PPP-P), PPP of lateral metatarsal (PPP-L), PPP of arch (PPP-A), PPP of heel (PPP-H).
- Indicators Related to the Center of Plantar Pressure (COP): Length of COP trajectory (COP-L), Maximum displacement of COP in the mediolateral direction (Max COP-ML)/ anteroposterior direction (Max COP-AP).

### Statistical analysis

Data analysis was performed using IBM SPSS Statistics 29.0. The normality of the data was tested by the Shapiro-Wilk test. Indicators that conformed to normal distribution were expressed as mean ± SD, while those that did not conform to normal distribution were expressed as M (IQR). When comparing the indicators among the four groups of subjects, the chi-square test was used for gender. For indicators that conformed to normal distribution and had homogeneous variance, One-way ANOVA was applied; for indicators that did not conform to normal distribution, the Kruskal-Wallis H Test was used, with Bonferroni correction. Then, Spearman correlation analysis was performed between each indicator and BMI. All statistical tests were conducted with a significance level of α = 0.05.

### Ethics approval and consent to participate

The study was conducted according to the guidelines of the Declaration of Helsinki and approved by the Ethics Committee of The First Affiliated Hospital of China University of Science and Technology (Approval Code: 2023KY086). Informed consent was obtained from all subjects involved in the study.

## Results

### Analysis of basic characteristics of subjects in each group

Descriptive statistics for the study participants are summarized in Table 2. No statistically significant differences were observed among the groups in gender, age, EU shoe size, or height (all *p* > 0.05). However, significant between-group differences were found in weight and BMI (*p* < 0.05).

**Table 2:** General information of subjects in each group

Groups	*N*	Age (y) ^a^	Height (cm) ^b^	Weight (kg) ^b^	BMI (kg/m^2^) ^a^	EU Size (1) ^a^
Underweight	14	20.50(5)	169.50 ± 8.55	50.65 ± 6.97	17.69(1.44)	40(2)
Healthy weight	24	24.00(4)	171.17 ± 8.35	62.56 ± 7.17	21.29(2.04)	39.5(5)
Overweight	14	22.00(6)	171.29 ± 5.31	77.02 ± 6.01	26.55(1.88)	42(2)
Obesity	10	24.00(4)	169.10 ± 5.63	84.05 ± 5.31	30.06(1.33)	40(5)
p		>0.05	>0.05	<0.05	<0.05	>0.05

^a^M (IQR)

^b^Mean ± SD

### Comparison of gait characteristics among subjects in each group

Gait parameter results across the four groups are presented in Table 3. Statistically significant differences (*p* < 0.05) were identified in eleven parameters: SL SLA, RSS, %SST, %DSPT, %SPT, and PPP-M, PPP-P, PPP-L, PPP-A, and PPP-H.

**Table 3:** Various kinematic and kinetic parameters

Groups	Underweight	Healthy weight	Overweight	Obesity	F/H	*P*
SL (cm) ^b^	133.86 ± 11.28	127.50 ± 7.50	127.68 ± 7.20	123.66 ± 9.11	2.96	0.04
SLA (1) ^b^	0.79 ± 0.06	0.75 ± 0.44	0.75 ± 0.35	0.73 ± 0.05	4.14	0.01
SS (cm/s) ^b^	121.97 ± 11.82	113.43 ± 12.50	115.81 ± 9.99	113.95 ± 7.40	1.88	0.143
RSS (s^− 1^) ^a^	2.47(0.40)	1.80(0.43)	1.51(0.26)	1.38(0.20)	42.9	<0.001
Cad (step/min) ^b^	54.89 ± 2.99	53.36 ± 4.15	54.45 ± 2.57	55.35 ± 2.33	1.12	0.35
GC (s) ^a^	1.09(0.07)	1.13(0.11)	1.09(0.10)	1.10(0.08)	1.77	0.38
%SST (%) ^b^	34.27 ± 1.79	33.89 ± 1.30	32.76 ± 1.15	31.91 ± 1.41	0.55	<0.001
%SPT (%) ^b^	34.26 ± 1.93	33.87 ± 1.38	32.66 ± 1.15	31.82 ± 1.56	3.72	<0.001
SA (°) ^b^	2.60 ± 0.52	2.31 ± 0.46	2.41 ± 0.56	2.16 ± 0.38	3.08	0.163
FRA (°) ^a^	23.09(17.10)	24.11(19.60)	26.24(15.10)	25.50(13.34)	7.4	0.49
MPFA (°) ^b^	66.17 ± 6.15	62.35 ± 6.46	62.81 ± 6.36	64.71 ± 7.54	14.41	0.33
MDFA (°) ^a^	-20.65(11.23)	-25.21(5.33)	-24.09(4.97)	-23.91(6.56)	7.08	0.29
PPP-M (N) ^a^	247.33(71.67)	277.64(119.98)	433.62(80.77)	345.87(137.57)	26.06	<0.001
PPP-P (N) ^b^	186.30 ± 71.43	246.15 ± 64.28	263.91 ± 90.56	262.27 ± 72.18	1.72	0.026
PPP-L (N) ^b^	147.35 ± 67.30	182.46 ± 53.71	199.15 ± 70.74	221.10 ± 59.96	0.44	0.033
PPP-A (N) ^a^	96.14(152.09)	104.03(77.08)	170.99(87.09)	217.43(127.54)	21.16	<0.001
PPP-H (N) ^b^	441.81 ± 127.50	504.09 ± 102.67	574.22 ± 141.98	607.52 ± 118.65	1.16	<0.001
COP-L (cm) ^b^	12.98 ± 0.75	13.00 ± 0.61	12.97 ± 0.77	13.05 ± 0.55	0.03	0.99
Max COP-ML (cm) ^b^	0.66 ± 0.12	0.57 ± 0.16	0.70 ± 0.14	0.60 ± 0.17	2.544	0.07
Max COP-AP (cm) ^b^	8.07 ± 0.62	8.04 ± 0.58	8.25 ± 0.43	8.02 ± 0.53	0.52	0.67

Figure caption: *SL *Step length, *SLA *Step length ratio, *SS *Step speed, *RSS *Relative step speed, *Cad *Cadence, *GC *Gait cycle time, *%SST *Percentage of single support time, *% DSPT *Percentage of double support phase time, *%SPT *Percentage of swing phase time, *SA *Step angle, *MPFA *Maximum plantar flexion angle, *MDFA *Maximum dorsiflexion angle, *FRA *Foot roll angle, *PPP-M *Peak plantar pressure of medial metatarsal, *PPP-P *Peak plantar pressure of phalangeal, *PPP-L *Peak plantar pressure of lateral metatarsal, *PPP-A *Peak plantar pressure of arch, *PPP-H *Peak plantar pressure of heel, *COP-L *Length of COP trajectory, *Max COP-ML *Maximum displacement of COP in the mediolateral direction, *Max COP-AP *Maximum displacement of COP in the anteroposterior direction

^a^M (IQR)

^b^Mean ± SD, *P*<0.05

**Fig. 4:**
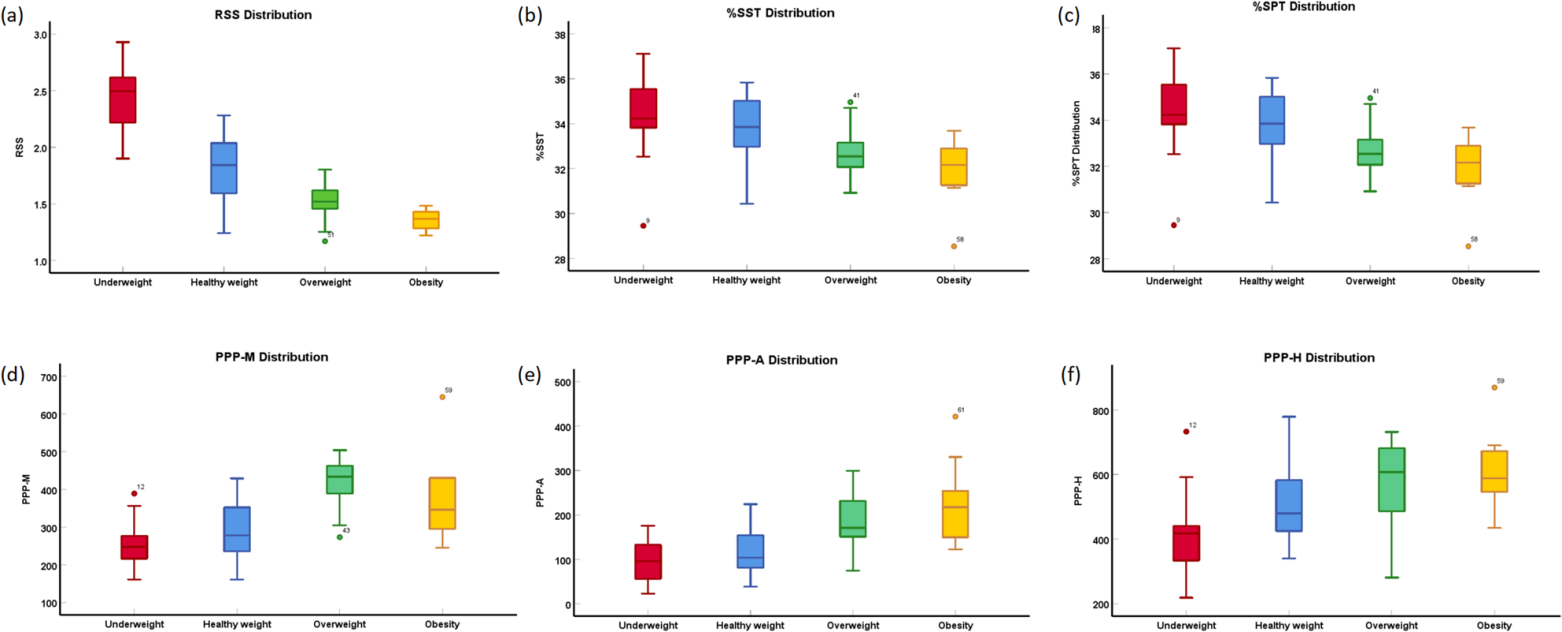
Box plot of the distribution of RSS (**a**), %SST (**b**), %SPT(**c**), PPP-M(**d**), PPP-A(**e**) and PPP-H(**f**) across different BMI groups (Underweight, Healthy weight, Overweight, Obesity). RSS=Relative step speed, %SST=Percentage of single support time, %SPT=Percentage of swing phase time, PPP-M=Peak plantar pressure of medial metatarsal, PPP-A=Peak plantar pressure of arch, PPP-H=Peak plantar pressure of heel

Following Bonferroni correction (significance level set at p' = 0.008), the distribution of these parameters is illustrated in Fig 4. A clear sequential trend was observed across underweight, normal weight, overweight, and obese groups: RSS, %SST, and %SPT progressively decreased, whereas PPP-M, PPP-A, and PPP-H progressively increased, with all trends being statistically significant (p < 0.001).

### Correlation analysis between BMI and gait parameters

**Fig. 5:**
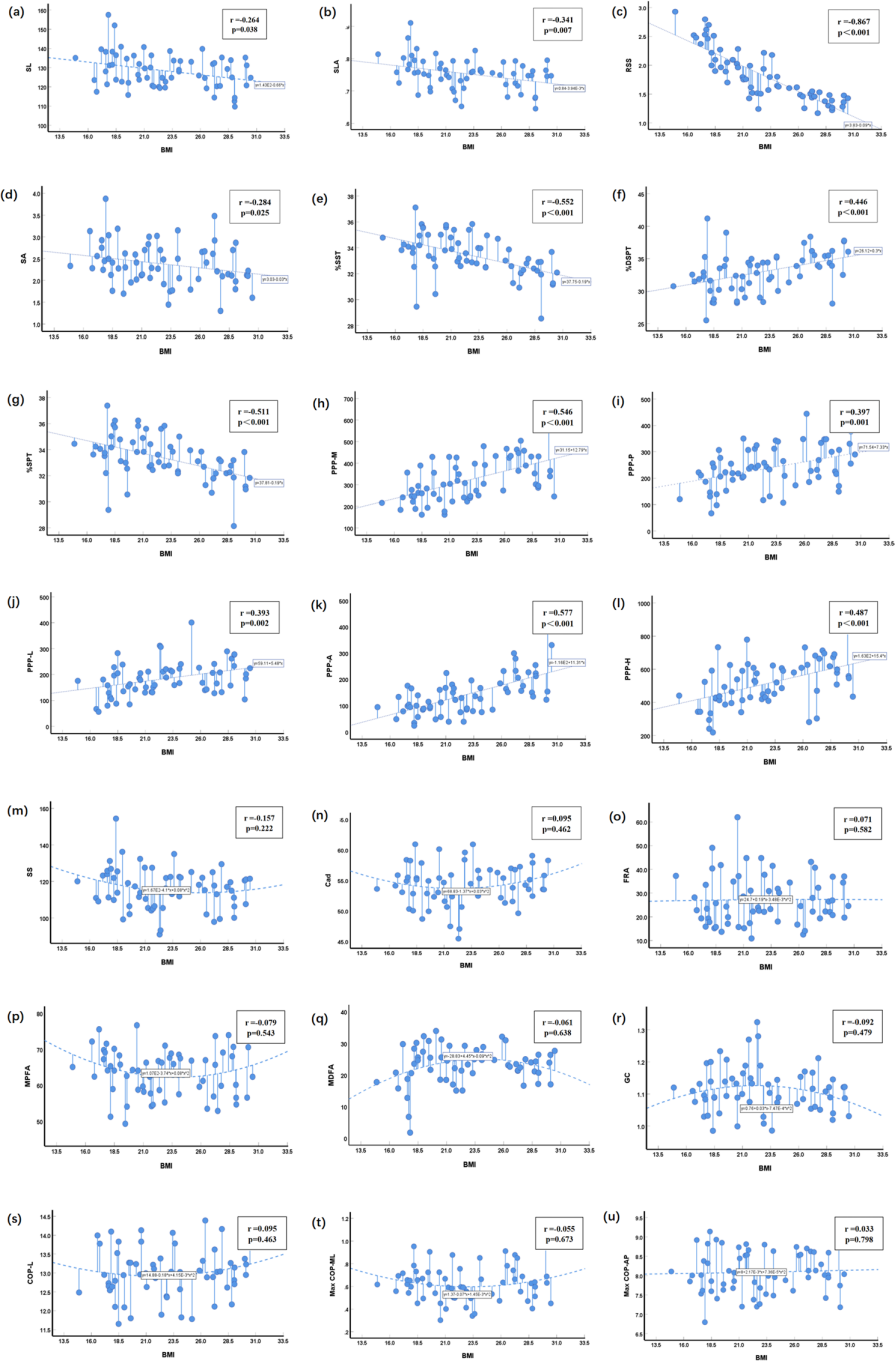
Shows the scatter plots of each index against the body mass index (BMI) and the results of Spearman correlation analysis. Specifically: In the scatter plots of SL (Step length) (**a**), SLA (Step length ratio) (**b**), RSS (Relative step speed) (**c**), SA (Step angle) (**d**), %SST (Percentage of single support time) (**e**), %DSPT (Percentage of double support phase time) (**f**), %SST (Percentage of single support time) (**g**), PPP-M (Peak plantar pressure of medial metatarsal) (**h**), PPP-P (Peak plantar pressure of phalangeal) (**i**) against BMI, the dotted line denotes the linear fitting curve; scatter plots of PPP-L (Peak plantar pressure of lateral metatarsal) (**j**), PPP-A (Peak plantar pressure of arch) (**k**), PPP-H (Peak plantar pressure of heel) (l), SS (Step speed) (**m**), Cad (Cadence) (**n**), FRA (Foot roll angle) (**o**), MPFA (Maximum plantar flexion angle) (**p**), MDFA (Maximum dorsiflexion angle) (**q**), GC (Gait cycle time) (**r**), COP-L (Length of COP trajectory) (**s**), Max COP-ML (Maximum displacement of COP in the mediolateral direction) (**t**), Max COP-AP (Maximum displacement of COP in the anteroposterior direction) (**u**)against BMI, the dotted line represents the curve fitting curve

Correlation analysis revealed several significant associations between BMI and gait parameters (Fig. 5):

- SL (*r* = -0.264, *p* = 0.038) and SLA (*r* = -0.341, *p* = 0.007) showed weak negative correlations with BMI.
- RSS (*r* = -0.867, *p* < 0.001) demonstrated a very strong negative correlation.
- %SPT (*r* = -0.552, *p* < 0.001) and %SST (*r* = -0.511, *p* < 0.001) were moderately negatively correlated with BMI.
- %DSPT (*r* = 0.446, *p* < 0.001) showed a moderate positive correlation.
- SA (*r* = -0.284, *p* = 0.025) was weakly negatively correlated.
- Peak plantar pressures showed positive correlations, being strong for PPP-M (*r* = 0.546) and PPP-A (*r* = 0.577), and moderate for PPP-P (*r* = 0.397), PPP-L (*r* = 0.393), and PPP-H (*r* = 0.487) (all *p* < 0.002).

No significant linear correlations were found between BMI and SS, Cad, MPFA, MDFA, GC, COP-L, Max COP-ML, or Max COP-AP. For parameters lacking significant linear correlation, we explored potential nonlinear relationships using parabolic regression (y = ax² + bx + c). The fitted curves revealed:

- An inverted U-shaped pattern (“high at both ends and low in the middle”) for Cad (cut-off: 22.83), MDFA (cut-off: 24.72), COP-L (cut-off: 21.69), and Max COP-ML (cut-off: 24.14).
- A U-shaped pattern (“low at both ends and high in the middle”) for GC (cut-off: 20.08) and MPFA (cut-off: 23.38).
- No consistent relationships were observed for SS, FRA, or Max COP-AP across BMI values.

## Discussion

This study utilizes a smart insole system to monitor gait parameters, providing new insights into the association between BMI and specific kinematic and dynamic gait characteristics.

### Adjustment of walking patterns from efficiency optimization to safety compensation

This study revealed that RSS of underweight individuals was significantly higher than that of other BMI groups, and RSS presented a very strong negative linear correlation with BMI (*p* < 0.001). This may be attributed to their lower body mass, which reduces exercise inertia and energy expenditure, facilitating easier limb initiation and swing. Furthermore, lower joint loads and swing resistance contribute to greater flexibility, which is also reflected in the weak negative linear correlations of SL and SLA with BMI (*p* < 0.05). However, underweight individuals often exhibit muscle fiber atrophy and reduced metabolic capacity, leading to diminished muscle strength and endurance, particularly in the dorsiflexor muscles [19]. To counteract body sway caused by muscular insufficiency during the support phase, they may unconsciously adopt a faster lower-limb swing to improve walking efficiency. This adaptation also results in a significantly higher percentage of single support time (%SST) and percentage of swing phase time (%SPT) (both *p* < 0.001), a lower percentage of double support phase time (%DSPT) (*p* < 0.05), which were moderately negatively and positively correlated with BMI, respectively.

In overweight individuals, excess body mass significantly increases lower-limb joint pressure and muscular energy consumption. Their gait demonstrates distinct adaptive adjustments consistent with the above BMI-dependent trends: to reduce the instantaneous impact force and muscular work per step, they actively shorten their SL, and the reduction in distance per step is not compensated by step speed (SS) or cadence (Cad), as no significant linear or nonlinear correlations were found between BMI and SS, Cad (*p* > 0.05). The single-leg support time is unconsciously shortened, while the double-support phase time is extended to facilitate a faster transfer of the center of gravity [20]. Similar adaptive gait characteristics have been observed in studies of the elderly [21]. Notably, multiple studies have not consistently identified clear gait parameter differences between young overweight and normal-weight groups [22, 23]. This may be because young people’s muscle strength, joint elasticity, and cardiopulmonary function are at their peak, which can compensate for the extra weight, and such subtle differences in RSS, %SST, %SPT and %DSPT can be accurately captured by smart insoles.

When body mass progresses to obesity, excessive load exacerbates joint pressure and cardiopulmonary burden, leading to altered skeletal muscle recruitment patterns (e.g., overactivation of the soleus and tibialis anterior during single support [24]), impaired contraction function (e.g., poor quadriceps function [6]), and structural changes (e.g., muscle fibrosis and structural disorganization [25]). To conserve energy and maintain stability, obese individuals further shorten their step length and reduce walking speed, thereby decreasing muscular work per unit time [26], while extending the %DSPT to gain a wider support base [27, 28]. Concurrently, chronic load compresses and thickens anterior ankle structures (e.g., tendons and ligaments), but no significant correlation was found between BMI and MPFA, MDFA or FRA [29] (*p* > 0.05), meaning the impact of BMI on ankle joint angle parameters lacks statistical evidence in this study.Although long-term load stimulation can induce lower-limb muscle hypertrophy and an increase in absolute strength, relative muscle strength (normalized to body weight) actually decreases [30]. While the aforementioned gait adjustments in obese individuals may reduce short-term joint load and improve stability, prolonged excessive muscle co-activation increases the risk of strain, and a shift in muscle fiber type compromises endurance [31]. This not only predisposes individuals to gait disorders and falls but also accelerates joint degeneration, increasing the incidence of conditions like osteoarthritis and tendinitis [32].

### The compensatory mechanism for phasic differences in plantar pressure

Our study identified statistically significant differences in PPP of all five foot regions among underweight, normal-weight, overweight, and obese groups (*p* < 0.05), and all PPP indicators presented positive linear correlations with BMI (*p* < 0.002). In particular, the PPP-M, PPP-A, and PPP-H were significantly higher in the overweight and obese groups compared to the normal-weight and underweight groups, and these three indicators showed a progressive increase with the rise of BMI (*p* < 0.001), which aligns with findings from Liu et al. [33]in elderly women and is likely related to weight-induced gait adaptations.

Body weight is the primary source of plantar pressure, and an elevated BMI significantly increases the body’s vertical load. During walking, different regions of the foot distribute this load. The heel, as the initial contact point, bears the brunt of the impact, and its PPP-H naturally rises with BMI [34], consistent with the conclusions of Choi et al. [31]. Sustained high heel pressure can lead to local pathologies like plantar fasciitis and Achilles tendinitis [10].The foot arch is a key structure for cushioning and distributing pressure. Prolonged compression from excess weight can lead to the relaxation of soft tissues like the plantar fascia, potentially resulting in arch collapse and valgus [35]. Following arch collapse, pressure in the metatarsal region increases accordingly [36], with the medial metatarsals being more affected [34]. This abnormal load distribution further impairs lower-limb alignment, causing excessive internal rotation of the knees and hips [37], which accelerates articular cartilage wear and increases the risk of osteoarthritis, leading to chronic tension and strain in lower-limb muscles [22, 38].

Notably, no significant linear correlations were found between BMI and all COP indicators (COP-L, Max COP-ML and Max COP- AP ). Although parabolic regression found an inverted U-shaped nonlinear relationship between Max COP-ML and BMI (cut-off: 24.14), this nonlinear trend lacks sufficient statistical power to confirm a definite association, and thus cannot be regarded as a BMI-dependent adaptive characteristic of the COP. The lateral metatarsal region is less affected by medial arch collapse, and the phalangeal region only bears short-term push-off loads [39]. Obese individuals compensate by shortening their step length and reducing push-off force, which mitigates inter-group pressure differences in this area. Nevertheless, increased body load can still cause injuries to the phalanges and soft tissues, including stress fractures [40].

In contrast to the high plantar pressure in individuals with high BMI (BMI > 24), our study shows that underweight individuals have generally lower peak plantar pressure than the normal-weight group, consistent with Shen et al. [41]. Biomechanically, excessively low plantar pressure indicates insufficient transmission of ground reaction forces to the lower limbs. Normal mechanical loading is a crucial stimulus for chondrocyte activity [6]. Chronic underloading may deprive cartilage of adequate stimulation, leading to reduced nutrient supply and slowed matrix synthesis, potentially triggering degenerative changes such as thinning and loss of elasticity [42]. Furthermore, underweight individuals often have insufficient nutrient intake, exacerbating deficiencies in bones, cartilage, and muscles. However, the hypothesis that this results in structural abnormalities and reduced tissue stability reflected in COP indicators is not supported by statistical results in this study, as no significant association was found between BMI and COP parameters.

Although the characteristics of some gait parameters and their mechanisms align with relevant studies, the inter-group differences in this study were not always statistically significant. This may be because the participants were young, with strong muscles, good joint elasticity, and flexible neural regulation, allowing them to compensate for weight differences through fine gait adjustments, thereby attenuating inter-group disparities.

### Limitations

This study utilized a smart insole system to monitor gait parameters, which provides novel insights into the association between BMI and specific kinematic as well as kinetic gait characteristics.

- Cross-sectional design and causality: The cross-sectional nature of the study only allows for the identification of correlations between BMI and gait parameters, not causal relationships. We cannot determine whether BMI changes induce gait adaptations or if inherent gait patterns influence weight regulation over time. Longitudinal studies tracking BMI and gait parameters over years are needed to clarify causal directions.
- Uncontrolled confounding factors: Key variables such as physical activity levels, exercise habits, shoe-wearing preferences, and foot morphology (e.g., arch height) were not systematically collected or controlled. These factors may independently influence gait parameters and plantar pressure distribution, potentially confounding the association between BMI and gait adaptations. For example, regularly active individuals with high BMI may have stronger lower limb muscles, leading to different gait compensatory mechanisms compared to sedentary individuals with the same BMI.
- Limited generalizability: The study population consists of healthy college students (18–28 years old) with no underlying musculoskeletal diseases. This homogeneous group has strong neuromuscular plasticity and compensatory capacity, which may differ from older adults, individuals with chronic conditions, or non-student populations. Thus, the findings cannot be directly generalized to these groups.

Simplified testing scenario: Gait data were collected during a standardized 10-meter linear walk, which does not reflect complex real-life scenarios (e.g., climbing stairs, turning, uneven terrain). Gait adaptations to BMI may differ in more dynamic environments, limiting the ecological validity of the results.

## Conclusion

Human gait is a complex motor behavior resulting from the dynamic balance achieved through integrated neural regulation, muscular force, and skeletal support. Using smart insole technology to analyze the gait parameters of college students across different BMI levels, this study found that several parameters showed significant differences and a consistent trend of change among underweight, normal-weight, overweight, and obese groups. These findings reveal the specific gait adaptation mechanisms the body employs to manage load and maintain balance across a mass gradient. This work provides a feasible technical method and quantitative indicators for the early screening of BMI-related gait abnormalities and for monitoring adolescent sports health.

## Supplementary Information

Supplementary Material 1

## Data Availability

The datasets generated and/or analyzed during the current study are not publicly available due to privacy and ethical restrictions but are available from the corresponding author on reasonable request.
